# Trait-based patterns of microbial dynamics in dormancy potential and heterotrophic strategy: case studies of resource-based and post-press succession

**DOI:** 10.1038/s41396-018-0194-x

**Published:** 2018-06-29

**Authors:** Patrick J. Kearns, Ashley Shade

**Affiliations:** 1 Department of Microbiology and Molecular Genetics, Michigan State University, East Lansing, MI USA; 2 Plant Resilience Institute, Michigan State University, East Lansing, MI USA; 3 Program in Ecology, Evolution, and Behavior, Michigan State University, East Lansing, MI USA; 4 DOE Great Lakes Bioenergy Research Center, Michigan State University, East Lansing, MI USA; 5 Department of Plant, Soil and Microbial Sciences, Michigan State University, East Lansing, MI USA

## Abstract

Understanding the relationship between microbial community structure and function is a major challenge in microbial ecology. Recent work has shown that community weighted mean 16S rRNA gene copies, as a proxy for heterotrophic growth strategy, is a microbial community trait that decreases predictably over successional trajectories that are underpinned by changes in resource availability. However, it has been challenging to identify other microbial traits that are predictive of community functions and have consistent patterns with succession. Trait-based patterns of secondary succession (e.g., after a disturbance) are less often considered, and these responses may be underpinned by abiotic drivers other than changes in resources. In this perspectives piece, we present hypotheses about microbial traits important for microbial succession in resource-based and post-press disturbance scenarios, as synthesized from previous works and extended within this work. Using four case studies, we compare two traits, heterotrophic strategy and dormancy potential, and two different types of succession, resource-based (endogenous heterotrophic) and post-press. There were decreases in weighted ribosomal operon counts and in dormancy genes over resource-based succession. Both traits also were lower in post-press succession as compared to reference conditions, but increased with time from disturbance. Thus, dormancy potential may be an additional trait that changes predictably with succession. Finally, considering changes in microbial community traits over post-press succession is as important as over resource-based succession. These patterns need to be interpreted carefully and reference and recovering samples can be collected to improve interpretation of changes in community traits over post-press succession.

## Approaching succession from the microbial perspective

Microbial succession includes two categories that have been borrowed from plant ecology: primary and secondary succession. These categories, however, do not fully capture the environmental context and physiology that distinguish microbial succession [1]. Fierer et al. [1] delineated microbial primary succession based on resource dynamics into three categories: autotrophic succession and endogenous or exogenous heterotrophic succession. Autotrophic succession occurs when early colonizers are primarily autotrophic and generate a stable, slow changing carbon pool over time. Heterotrophic succession is dictated by the source of carbon and the early colonization of heterotrophic taxa. Endogenous succession relies on the respiration of local carbon and succession is driven by changes in the carbon pool (e.g., as in colonization of a nutrient-rich mesocosm; Nemergut et al. [2]). Exogenous succession relies on the resupply of external carbon and its variabile amount and quality. All three types of “primary” succession, however, are dictated by changes in resources and as such, we broadly refer to these as *resource-based succession* (Table 1). Fierer et al. [1] also specified that these types of microbial succession initiate from a “blank-slate” environment that was either sterile or nearly sterile, analogous to primary succession in plants.

**Table 1:** Characteristics of microbial succession and their relationships to concepts in plant ecology and microbial ecology

Term used in this study	Resource-based succession	Post-disturbance succession
Microbial ecology terms	e.g, Autotrophic, endogenous heterotrophic, exogenous heterotrophic [1]	E.g., post-press, post-pulse (this work)
Plant ecology term	Primary	Secondary
Initial environment	Sterile/near sterile	Not sterile/previously colonized
Primary driver	Resource changes	Disturbance, indirect drivers, e.g., plants, pH
Trophic progression	Copiotrophic to oligotrophic	Oligotrophic to oligotrophic or oligotrophic to copiotrophic expected for most soils, but will depend on the pre-disturbance conditions
References	Fierer et al. [1], Nemergut et al. (2015)	This work
Case studies analyzed here	Ferrenberg et al. [10]: forest fire-affected bacterial communities and the subsequent recovery of these communities. Nemergut et al. [2]: shifts in rrn copy number in 4 nutrient-based succession studies	DeAngelis et al. [9]: mild warming affected bacterial communities after 20 years. Lee et al. [11]: shifts and subsequent recovery of bacterial communities in response to an underground coal fire. Aanderud et al. [24]; Nemergut et al. [2]; Yano et al. [20]

For a conceptual model of these patterns based on the datasets included in this study, please see Fig. 3

In contrast to resource-based succession, microbial “secondary” succession occurs following a disturbance to a previously colonized ecosystem. In ecology, secondary successional patterns can depend on new immigrants that colonize the disturbed ecosystem, but local taxa can also play an important role. Local taxa that persist despite the disturbance and/or gain a competitive advantage given the disturbance can affect community outcomes. Thus, resuscitated microbial taxa may contribute substantially to microbial secondary succession, which may be a point of distinction from “macrobial” succession (e.g., see Nemergut et al. [3]). Local taxa that have historically or contemporarily contributed to the dormant pool provide an opportunity for legacy effects of previously successful community members [4]. Furthermore, resuscitation can allow for the proliferation of taxa that were not competitive before the disturbance. Thus, the dynamics of secondary succession in plants are considerably different than those of microbes, and bacterial and archaeal dynamics may be more influenced by the local source pool, rather than immigration of new taxa. Furthermore, post-disturbance microbial succession is not necessarily driven by changes in resources, but instead by resistance and resilience to the stressor by persisting populations. Because of these distinction between microbes and plants, we offer a re-focusing of microbial secondary succession to *post-disturbance succession*, which can be further delineated into *post-press* (after a long-term disturbance that impacts multiple generations) and *post-pulse* (after short-term disturbance) disturbance scenarios (Table 1).

## Microbial community traits that change with succession

The succession of microbial communities following a disturbance can have important implications for the recovery and maintenance of ecosystem function [5]. Two potentially important microbial traits are dormancy potential and the number of ribosomal operons (hereafter “operon count”). Dormancy is the ability of microorganisms to decrease metabolic activity and maintain viability in a quiescent state (e.g., see ref. [6]), and it has implications for a microorganism’s ability to persist in the environment given unfavorable conditions. Operon count is the number of ribosomal operons within a cell, and has been used as a proxy for a microorganism’s heterotrophic strategy and therefore the rapidity of its response to resources; copiotrophs are assumed to have relatively more copies than oligotrophs [7]. While rapid growth and operon count have been shown to be correlated in laboratory cultures of type strains [8], there is limited information about how the growth strategies of most environmental taxa relate to ribosomal operon count, especially in situ. However, mean weighted ribosomal operon count across taxa, as assessed by 16S rRNA gene amplicon sequencing followed by metagenome reconstruction, has been introduced as an aggregate microbial community-level trait for heterotrophy (see Nemergut et al. [2], and ref. [9]).

## Case studies

We explored the patterns of two traits, ribosomal operon count and dormancy potential (measured as the abundance of genes conferring dormancy strategies), over microbial community succession in four previously published studies, three involving soils (Table S1). Two studies were examples of endogenous heterotrophic succession over changes in resource availability [10] (Nemergut et al. [2]). In addition, we investigated two sites exposed to mild and extreme increased temperatures as examples of post-press succession (see refs. [9, 11], respectively).

The studies of Ferrenberg et al. [10] and Nemergut et al. [2] are examples of succession driven by changes in type and availability of resources after colonization of a “blank-slate” environment [1]. Nemergut et al. [2] examined community succession over a 96-h period in sterilized rich media mesocosoms deployed in a coastal forest on the Yucatan Peninsula, Mexico. Ferrenberg et al. [10] collected samples following a forest fire on the eastern slope of the Colorado Front Range, CO, USA. The top 5 cm of soils were collected at reference sites and at a fire-affected sites at 1, 4, 29, and 33 months post fire disturbance. While this study would be classified as secondary succession based on the plant literature, we posit that, from the microbial perspective, the forest fire study more closely resembles endogenous heterotrophic (resource-based) succession for the soil microbial communities, as distinguished by Fierer et al. [1]: the top 5 cm of collected soils were likely sterilized from the fire (a “blank-slate” environment), and there were reported changes in organic matter quality (lower C:N ratio) and other important nutrients (higher NH_4_^+^) [10], suggesting that the trajectory was primarily driven by the dynamics of available resources. It was previously reported by Nemergut et al. [2] that weighted mean operon count decreased over succession in both of these studies, suggesting a gradual replacement of copiotrophic colonizers with oligotrophs.

The studies from DeAngelis et al. [9] and Lee et al. [11] are examples of post-press succession studies in soils following heat disturbance. The study by DeAngelis et al. [9] examined the effect of increased temperature (+5 °C) on temperate forest soils (Harvard Forest LTER, Petersham, MA, USA) after 5, 10, or 20 years of warming. Soils were collected from the O (0–0.03 m) and A (0.03–0.13 m) horizons. The authors demonstrated a decrease in weighted mean operon count in heated O horizon soils relative to reference soils but found no change in the A horizon. They also reported no difference in operon count given different durations of warming. Thus, we focused on O horizon soil communities and aggregated over years of warming. Finally, Lee et al. [11] examined a chronosequence of surface soil impacted at different decades by the progression of the Centralia underground coal seam fire (Pennsylvania, USA). The fire underlies 150 acres of temperate forest and remaining town, and warms the surface soil (fire-affected temperatures ranged from ~20 to 60 °C). Samples were collected from the top 20 cm of soil from unvegetated sites that were fire-affected, recovered from fire, and reference. The original study did not analyze weighted mean operon count.

## Results and discussion

For each study, we calculated weighted mean operon count by summing the relative abundance of each taxa multiplied by its copy number as determined by PICRUSt [12], replicating the previous analyses of copy number as a community-level aggregated trait [2, 9]. We first reproduced the analyses that showed that operon count decreased over resource-based succession (Fig S1A-B; Nemergut et al. [2]). In agreement with the previous reports, and, as expected, operon count decreased over succession with colonization of the sterile mesocosms and soils that had more recently experienced fire (4 months recovered) had higher operon counts than soils that were further removed from the time of disturbance (29 months recovered). This agrees with the previously posed hypothesis of copiotroph colonizers followed by oligotroph successors during resource-based primary succession [2].

**Fig. 1:**
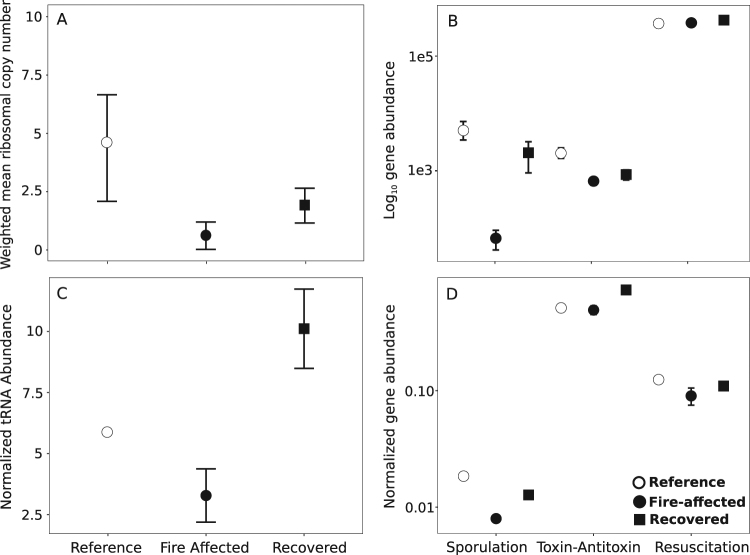
Two microbial traits, ribosomal operon count and dormancy potential, are decreased in fire-affected Centralia soils relative to recovering and reference soils. Plot of weighted mean ribosomal copy number (**a**) and log10 abundance of dormancy genes (**b**) in Centralia soils as estimated by PICRUSt, and metagenomic analysis of relativized tRNA abundance (**c**) and dormancy gene abundance (**d**). Relativized tRNA abundance is used in place of 16S rRNA operon count due to the difficulty in assembling rRNA and the high correlation between tRNA and rRNA abundances. Points are means and error bars are standard error of the mean. Note differing scales between **a**–**d**

We next reproduced the analysis that showed that operon count after experimental long-term soil warming at Harvard Forest had higher operon count in reference soils than in warmed soils, as an example of post-press succession (Fig S1C; Kruskal–Wallis test, *p* < 0.001, *H* = 19.38). We then added an analysis of our own published dataset of post-press succession in Centralia. In Centralia, fire-affected soils had lower operon count than recovered soils, which had lower operon count than reference soils (Fig. 1a; *p* = 0.002, *H* = 12.07). This suggests that, over post-press succession, operon count decreases at/during disturbance and then increases during recovery. Thus, relative to reference soils, post-press succession can exhibit an opposite pattern than resource-based succession. An interpretation of this may be that the relative number of copiotrophs increases with time from disturbance in this scenario. During post-press succession, it may be that operon count patterns are conditional on (1) persistence of some members of the local community given the disturbance (e.g., an unsterile starting environment and the local pool of dormant organisms); (2) the contribution of important drivers other than changes in resource quality and availability; and (3) competitive differences in the community members to the disturbance, resulting in differential survivorship and proliferation.

Overall, these results agree with previous studies: operon count is an aggregated community trait that can inform patterns of microbial succession. However, we show that operon count patterns over resource-based and post-press succession can be different. A more nuanced interpretation of operon count dynamics may be necessary to inform drivers of post-press succession, and a specific consideration of the conditions of resource-based succession (exogenous/endogenous and autotrophic/heterotrophic), driven by changes in resource availability from a “blank-slate” environment [1] will be informative for predicting trait-based outcomes. Furthermore, the correlation between copiotrophy and ribosomal operon count is still unclear and investigations into other genomic features such genomic architecture (e.g., position of genes) or genome size may provide a more complete picture [7, 13].

**Fig. 2:**
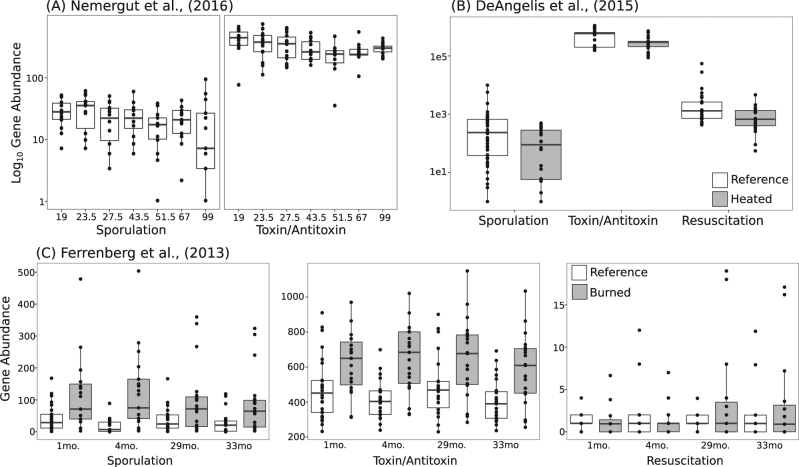
Genes underlying dormancy strategies generally decrease during resource-based (**a**, **c**) and post-press succession (**b**). Dormancy genes (sporulation factors, toxin–antitoxin systems, and resuscitation-promoting factors) were estimated using PICRUSt. Numbers above boxes in (**a**) show the times the mesocosms were sampled (h). No resuscitation-promoting factors were found in (**a**). Note the differing *y*-axis ranges between panels

Next, we assessed the patterns of microbial pathways involved in initiating or regulating microbial dormancy [6]. We focused on: sporulation factors (*spo* genes) that are generally conserved among Firmicutes [14]; toxin–antitoxin systems (hipA/B, MazF/E, RelB/E, and DinJ/YafQ) that are phylogenetically distributed among Gram-positive bacteria, Gram-negative bacteria, and archaea [15] and commonly detected in metagenomes [6]; and resuscitation-promoting factors (*rpfC*) that are conserved among Actinobacteria with homologs among some Firmicutes [16]. While these are not an exhaustive set of dormancy genes, they represent the major known strategies and lineages of microbes capable of dormancy [6]. We used PICRUSt to reconstruct metagenome content for each study and queried these for dormancy genes. Over primary succession, there were general decreases in dormancy genes over time (Fig. 2a, b). In the forest fire dataset, post-fire soils had more dormancy genes than reference soils (Fig. 2b; *H* = 8.23, *p* = 0.004). Over secondary succession, there were relatively more dormancy genes in reference and recovered soils as compared to fire-affected soils in Centralia (Fig. 1b; *H* = 41.093*, p* < 0.01) and to warmed soils in the Harvard Forest (Fig. 2c; *H* = 198.02, *p* < 0.01). Inclusive of all studies, there was a positive relationship (Spearman’s *ρ* = 0.44–0.78, *p* < 0.001) between weighted mean operon count and dormancy gene abundance, suggesting a possible link between copiotrophy and potential for dormancy. Thus, we analyzed publicly available bacterial genomes to determine if there was a relationship between operon counts and dormancy potential (assessed using rrnDB [17]). We found that genomes with more ribosomal operons were likely to also contain these dormancy genes (Fig S2; *H* = 1326.6, *p* < 0.01).

There are limitations in using metagenome reconstruction from 16S rRNA gene amplicon libraries and some discussion about the accuracy of ancestral state reconstruction for environmental microorganisms that are not well represented in genome databases [12]. Thus, we performed complementary analyses to estimate ribosomal operon counts and dormancy genes using annotated metagenomes that were also sequenced from Centralia soils. We used the abundances of the transfer RNA (tRNA) genes instead of 16S rRNA genes due to the difficulties in assembling 16S rRNA genes. There was a strong correlation between 16S rRNA and tRNA abundance (*r*^2^ = 0.8 and 0.96 in our dataset), as previously reported [17]. Additionally, we analyzed operon count in Centralia soils using the ribosomal operon database (rrnDB[17]), and there was a strong correlation (*ρ* = 0.86, *p* < 0.01) between ribosomal operon counts estimated by PICRUSt and the rrnDB (Fig S3).

Normalized tRNA abundance was significantly higher in recovered soils than fire-affected soils (Fig. 1c; *F* = 127.19, *p* = 0.005). This suggests that operon count per genome was decreased due to the fire and that fewer copiotrophic bacteria were present. We also observed more dormancy genes in reference and recovered soils than in fire-affected soils (Fig. 1d; toxins *F* = 4.13, *p* = 0.04, sporulation *F* = 27.18, *p* < 0.01); however, no significant effect was found for resuscitation-promoting factors (*F* = 1.82, *p* = 0.07). Our results show an unexpected agreement in pattern between metagenome analysis, rrnDB analysis, and metagenome reconstruction from 16S rRNA gene sequences (Fig S3, Fig. 1c). This suggests that though databases may be limited, the metagenome patterns derived from 16S rRNA gene sequences were robust across multiple methodologies.

The results presented here suggest nuances in patterns of ribosomal operon count between resource-based and post-press microbial succession. In resource-based succession, fast growers with high ribosomal operon count are favored by the high resource availability in early succession (rich media and new resource availability following forest fire). Furthermore, early colonizers also had higher potential for dormancy, as assessed by dormancy gene abundances. Recent work suggests that many microorganisms have limited long-range dispersal capabilities [18], and that colonization of a blank-slate environment likely occurs from regional metacommunities. The mesocosms investigated by [2] had a diversity of colonizers. However, there was consistent detection of taxa from the endospore-forming Firmicutes phylum when nutrients were high. The early mesocosm colonization of taxa with dormancy potential may be reflective of the general hardiness and high dispersal potential of dormant cells [19], and their ability to grow efficiently [20].

In contrast to the patterns following resource-based succession, post-press succession case studies had a decrease in ribosomal operon count and dormancy traits with time and relative to reference soils. Increased temperature directly stresses cells and alters soil biogeochemistry. In Centralia, extreme temperatures impose a harsh environment that may also favor oligotrophic growth. Though we do not know how representative they are, the post-press succession case studies presented suggest an overall reduction in microbiome dormancy potential after a press stressor. This is important because dormancy has been linked to the preservation of ecosystem function following disturbance [21, 22] and it suggests lower community resilience to future stressors. Data from the recovered soils in Lee et al. [11] suggest partial recovery of dormancy genes following release of the stressor. A next step would be to determine whether the partial recovery of dormancy genes can be attributed to immigration from the regional species pool. Nonetheless, while dormant taxa and rare microbial taxa may provide reservoirs of microbial diversity and function [5], we propose that the loss of dormancy potential can alter subsequent post-disturbance successions and microbial functional responses to future disturbances.

Though, in some cases, dormancy genes and operon counts were positively correlated, we do not expect this to be universal for all microorganisms and ecosystems. The observed relationship between dormancy gene abundance and operon counts may be due to the general phylogenetic conservation of some of the dormancy genes (e.g., s*po* genes and *rpf*), as operon count often is also conserved or similar within lineages [17]. While the operon counts of genomes containing toxin–antitoxin genes was higher than those from genomes in which no dormancy genes were detected (*p* < 0.01, *H* = 1326.6), the overall correlation between toxin–antitoxin systems and operon count was low relative to the other dormancy genes (Spearman’s *ρ* = 0.44 compared to 0.78). Among the dormancy genes investigated here, toxin–antitoxin genes are most phylogenetically broad and least specific to dormancy strategies (e.g., involved in other pathways), suggesting that dormancy potential is not necessarily linked to operon count or heterotrophic strategy in all situations. We are yet unable to fully catalog this trait because of limitations in annotation of divergent, and novel dormancy genes. An improved understanding of the phylogenetic conservation [23] of dormancy genes will inform their relationship with heterotrophic strategy.

**Fig. 3:**
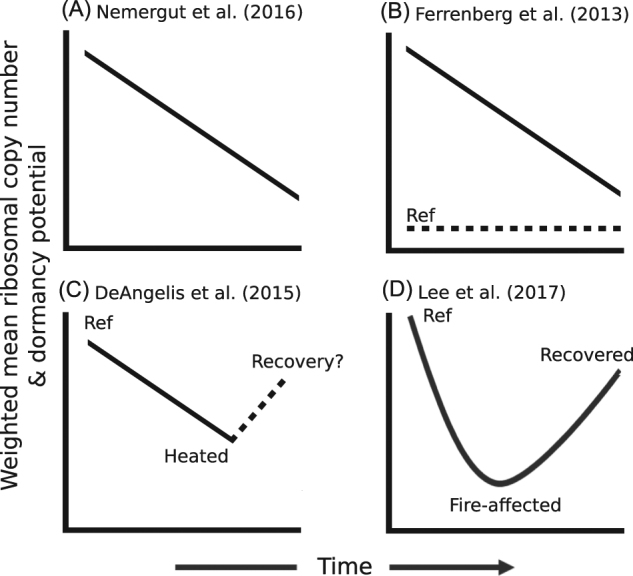
Schematic of the dynamics of microbial traits in case studies of endogenous resource-based (**a**, **b**) and post-press (**c**, **d**) succession. All studies had decreases in ribosomal operon count and dormancy potential after disturbance, but the patterns were different with respect to reference soils. Specifically, operon counts and dormancy gene abundances over post-press succession studies were lower relative to reference, while they were higher in resource-based succession

In investigating patterns of post-press succession, informative comparisons are made to reference dynamics and recovered conditions. Operon count and dormancy gene abundance did not return to reference levels after 33 months of recovery in [10] (Fig. 3). However, both post-press succession studies had a lower abundance of these traits relative to reference soils. Though data from Lee et al. [11] indicate a partial recovery of traits following stressor release, the degree of recovery after mild soil warming is unknown yet [9]. We highlight the need for observation of reference communities to better understand the dynamics occurring during succession, and for inclusion of recovery time points to fully understand long-term trait dynamics and their associated ecosystem functions. Post-disturbance succession, whether pulse or press, may necessarily be more nuanced towards disturbance characteristics and its specificity to hinder or advantage the growth of certain populations. For example, changes in community structure due to temperature increases will not be the same as changes due to salinity or pH, but in combining case studies, it may be possible to observe overarching patterns in the traits of taxa both sensitive and tolerant to disturbances.

In conclusion, we have presented a revised conceptual framework for microbial succession and four case studies to suggest that, in addition to weighted ribosomal operon count, dormancy potential is a microbial trait that could be useful for interpreting nuanced patterns of microbial succession. Because they may enhance ecosystem stability via member persistence, taxa that employ dormancy strategies likely play key roles in post-disturbance succession. In addition, regional taxa that employ dormancy strategies robust to dispersal may serve as important pioneers in resource-based succession. The case studies here can speak only to endogenous heterotrophic succession, but autotrophic and exogenous heterotrophic succession may benefit from initially dormant pioneers as well. More synergistic analyses of studies are needed to understand the generalities of microbial succession, including autotrophic, exogenous heterotrophic, post-press, and post-pulse scenarios. Ultimately, linking changes in these and other microbial traits to changes in function will allow for improved prediction of ecosystem outcomes over both resource-based and post-press succession.

## Electronic supplementary material

Supporting Information
