# Supplementary material for: Trait-based patterns of microbial dynamics in dormancy potential and heterotrophic strategy: case studies of resource-based and post-press succession
Source: ISME J. 2018 Jun 29;12(11):2575–81. doi: 10.1038/s41396-018-0194-x (PMC6194022; doi:10.1038/s41396-018-0194-x)
Supplement: Supplementary file 1 — Supporting Information [file 41396_2018_194_MOESM1_ESM.docx]

Supporting Information for

Trait-based patterns of microbial dynamics in dormancy potential and heterotrophic strategy: case studies of resource-based and post-press succession

By Patrick J. Kearns and Ashley Shade

**Supporting Methods**

***16S rRNA gene amplicon analysis***

Paired-end reads from Lee and Sorensen et al., (2017) were joined and quality filtered with a partial UPARSE pipeline (Edgar, 2013) as described in that work. Data from DeAngelis et al., (2015), Ferrenburg et al., (2015), and Nemergut et al., (2015) were acquired in FASTA format and no additional quality filtering was performed beyond what the authors previously reported. Data from each study was clustered at 97% identity with uclust (v. 8.0; Edgar, 2010) against the 13.5 version of GreenGenes (DeSantis et al., 2006). The resulting OTU tables were uploaded to the Galaxy web server (https://huttenhower.sph.harvard.edu/galaxy/) and analyzed with PICRUSt (Langille et al., 2013). OTU tables were first normalized by ribosomal operon copy number before metagenome prediction. We calculated weighted mean copy number as described previously (Nemergut et al., 2015) by summing the relative abundance of each taxa multiplied by its copy number. Because the release date of the most recent GreenGenes database was from May 2013, we also assessed operon counts in the Centralia dataset by analyzing our data with the Ribosomal RNA Database (rrnDB; Stoddard et al., 2015). We uploaded representative OTU sequences to the rrnDB and used the estimated copy number for each OTU to calculate weighted mean copy number as described above. From the PICRUSt-estimated metagenomes, we focused on genes associated with dormancy pathways using the KO identifiers K07699 (*spo0A*), K10715 (*rpfC*), K07154 (*hipA*), K03830 (*yafP*), K07172 (*mazE*), K06218 (*relE*), K01451 (*hipO*), K07473 (*dinJ*), K07171 (*mazF*), and K00951 (*relA*). Significant differences in the abundance of dormancy genes and operon counts were determined with either an ANOVA or a Kruskal-Wallis test in R (R Development Core Team).

***Metagenome analysis of Centralia soils***

For the Centralia metagenomes, DNA was extracted using a phenol-chlorofom method (Cho et al., 1996) and purified with the MoBio DNEasy PowerSoil Kit (MoBio, Carlsbad, CA, USA) following the manufacturer’s instructions. Metagenomic sequencing was performed by the Department of Energy’s Joint Genome Institute (DOE JGI) on an Illumina HiSeq 2500. Assembly and processing of raw reads were processed following JGI’s standard operating procedures (http://www.**jgi**.doe.gov). Annotated dormancy genes were retrieved from IMG using KO identifiers K07699 (*spo0A*), K10715 (*rpfC*), K07154 (*hipA*), K03830 (*yafP*), K07172 (*mazE*), K06218 (*relE*), K01451 (*hipO*), K07473 (*dinJ*), K07171 (*mazF*), and K00951 (*relA*). tRNA and dormancy gene abundance were normalized to the single copy house-keeping gene *rplB*.

**Data and workflow availability**

16S rRNA gene data from each study was collected from either NCBI, FigShare, or through personal communication with the study’s authors. Lee and Sorensen et al., (2017): NCBI SRA SRP082686, DeAngelis et al., (2015): NCBI SRA SRP040706, Nemergut et al., (2015): <https://doi.org/10.6084/m9.figshare.1556152.v1>, and data from Ferrenburg et al., (2013) was acquired through personal communication with a co-author from this study (Joseph Knelman). Centralia metagenomes are available at JGI IMG (GOLD Study ID Gs0114513) and computational workflows for analysis of PICRUSt and operon counts (<https://github.com/ShadeLab/Centralia_operons_dormancy)> and quality filtering of Centralia 16S rRNA gene data (<https://github.com/ShadeLab/PAPER_LeeSorensen_ISMEJ_2017>) are available on GitHub

**References**

Edgar RC. (2013). UPARSE: highly accurate OTU sequences from microbial amplicon reads. *Nat Methods* 10: 996.

Edgar RC. (2010). Search and clustering orders of magnitude faster than BLAST. *Bioinformatics* 26: 2460-2461.

DeSantis TZ, Hugenholtz P, Larsen N, Rojas M, Brodie EL, Keller K, et al. (2006). Greengenes, a chimera-checked 16S rRNA gene database and workbench compatible with ARB. *Appl Environl Microbiol* 72: 5069-5072.

Langille MG, Zaneveld J, Caporaso JG, McDonald D, Knights D, Reyes JA, et al. (2013). Predictive functional profiling of microbial communities using 16S rRNA marker gene sequences. *Nat Biotech* 31: 814-821.

Stoddard SF, Smith BJ, Hein R, Roller BR, Schmidt TM. (2014). rrn DB: improved tools for interpreting rRNA gene abundance in bacteria and archaea and a new foundation for future development. *Nucleic Acids Res* 43: D593-D598.

**Supplemental Figures**

**Figure S1. As a community-level microbial trait linked to heterotrophic strategy, weighted mean community ribosomal copy number decreased over time in a nutrient-rich mesocosm experiment (A), increased relative to reference soils during resource-based succession (B), and decreased relative to reference soils during post-press succession (C).** Weighted mean ribosomal gene copy number was calculated from 16S rRNA gene surveys for nutrient-based succession studies (A) Nemergut et al., (2015) and (B) Ferrenburg et al., (2013) and for the post-press succession study from DeAngelis et al., (2015) (C).


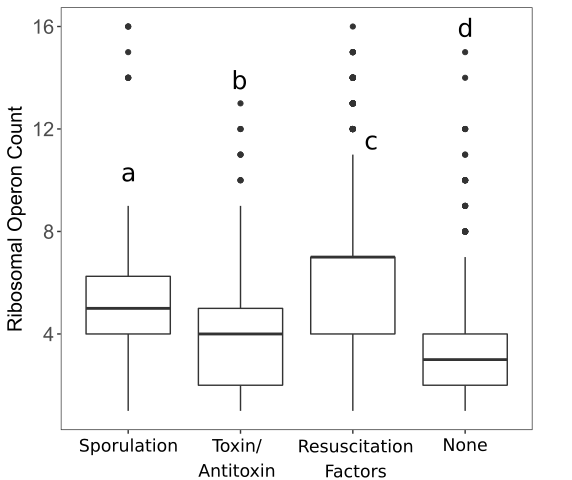


**Figure S2. The number of ribosomal operons in cultivated bacteria is higher for taxa with dormancy strategies.** Ribosomal operon counts for genomes in NCBI. The category ‘none’ refers to taxa without a significant BLASTn hit for any of the three dormancy strategies examined here. Letters indicate groups that are significantly different based on a Kruskal-Wallis test with a Dunn Test for multiple comparisons.

**Figure S3. There is agreement between methods to estimate ribosomal operon count based on 16S rRNA amplicon data.** Biplot of weighted mean ribosomal operon count estimated using PICRUSt and the ribosomal operon database. Datasets display a strong correlation (ρ=0.86, *p*<0.01).

Supporting Tables

Table S1- Case studies analyzed in this piece.

|  | Ferrenberg et al., (2013) | Nemergut et al., (2015) | DeAngelis et al., (2015) | Lee and Sorensen et al., (2017) |
| --- | --- | --- | --- | --- |
| Major driver of succession | Resource availability and changes | Resource availability and changes | Modest temperature increase driving biogeochemical changes | Extreme temperature increase driving biogeochemical changes |
| Succession Type | Endogenous heterotrophic, nutrient-based, (primary, post- sterilization) | Endogenous heterotrophic, nutrient based, (primary, “blank slate”) | Post press disturbance (secondary) | Post press disturbance (secondary) |
